# Cricket Oil-Based Sunscreen Systems: Formulation Design, Ultraviolet Protection Performance, and Preclinical Safety Evaluation

**DOI:** 10.3390/pharmaceutics18030325

**Published:** 2026-03-04

**Authors:** Wantida Chaiyana, Guijun Liang, Jirasit Inthorn, Pratthana Chomchalao

**Affiliations:** 1 Department of Pharmaceutical Sciences, Faculty of Pharmacy, Chiang Mai University, Chiang Mai 50200, Thailand; 2 Center of Excellence in Pharmaceutical Nanotechnology, Faculty of Pharmacy, Chiang Mai University, Chiang Mai 50200, Thailand; 3 Multidisciplinary and Interdisciplinary School, Chiang Mai University, Chiang Mai 50200, Thailand; 4 Research Center of Deep Technology in Beekeeping and Bee Products for Sustainable Development Goals (SMART BEE SDGs), Chiang Mai University, Chiang Mai 50200, Thailand; 5 School of Cosmetic Science, Mae Fah Luang University, Chiang Rai 57100, Thailand; 6331701501@lamduan.mfu.ac.th; 6 Department of Health and Cosmetic Product Development, Faculty of Food and Agricultural Technology, Pibulsongkram Rajabhat University, Phitsanulok 65000, Thailand; jirasit.i@psru.ac.th; 7 College of Medicine and Public Health, Ubon Ratchathani University, Ubon Ratchathani 34190, Thailand; pratthana.c@ubu.ac.th

**Keywords:** insect, cricket, oil, sustainable, ultraviolet protection, cell, safety, antioxidant, anti-inflammation

## Abstract

**Background/Objectives:** Insect oils have gained attention as sustainable cosmetic ingredients due to their bioactive lipid content. This study aimed to characterize oils from cricket and to evaluate their safety, biological activities, and performance in sunscreen formulations. **Methods:** Oils were extracted from *Gryllus bimaculatus*, *Teleogryllus mitratus*, and *Acheta domesticus* by cold pressing following hot-air drying. Fatty acid composition was determined using gas chromatography–mass spectrometry. Safety was assessed by cytotoxicity testing in normal human dermal fibroblasts (NHDF) and the hen’s egg chorioallantoic membrane (HET-CAM) assay. Antioxidant and anti-inflammatory activities were evaluated by intracellular reactive oxygen species (ROS) and nitric oxide (NO^•^) assays. Based on biological performance, *T. mitratus* oil (TMO) was incorporated into sunscreen creams containing physical and chemical ultraviolet (UV) filters. Physical stability, viscosity, pH, sun protection factor (SPF), persistent pigment darkening/ultraviolet A protection factor (PPD/UVA-PF), and blue light protection were evaluated. **Results:** All cricket oils were non-cytotoxic to NHDF cells and were classified as non-irritating in the HET-CAM assay. TMO exhibited the strongest antioxidant activity, reducing intracellular ROS and significantly inhibiting NO^•^ production in lipopolysaccharide-stimulated cells. Only TMO showed measurable UVA protection (PPD/UVA-PF = 12.1, PA+++). Sunscreen creams formulated with TMO achieved higher photoprotective efficacy than olive oil-based creams, with SPF values up to 40.51 and PPD/UVA-PF up to 39.17. The inclusion of foundation pigments further increased SPF to 43.09 and enhanced blue light protection to 35.1%. **Conclusions:** TMO is a safe and effective multifunctional ingredient that enhances sunscreen performance and supports sustainable cosmetic formulation.

## 1. Introduction

Solar radiation comprises ultraviolet (UV), visible, and infrared wavelengths, with ultraviolet A (UVA, 320–400 nm) accounting for approximately 95% and ultraviolet B (UVB, 280–320 nm) for about 5% of the UV radiation reaching the Earth’s surface [1]. The depth of skin penetration increases with wavelength: UVB radiation is limited to the epidermis, UVA penetrates the dermis, while visible (400–700 nm) and infrared radiation (over 700 nm) can reach the subcutaneous tissue [1]. UV exposure affects the skin through multiple mechanisms beyond sunburn and carcinogenesis [2]. UVB radiation is primarily responsible for acute skin responses such as sunburn and can directly damage deoxyribonucleic acid (DNA) and cellular proteins, whereas UVA radiation penetrates deeper into the dermis, where it promotes collagen degradation, elastin damage, and long-term photoaging, contributing to premature skin aging [2,3]. In addition, high-energy visible light, particularly blue light (400–500 nm), has recently been recognized as an emerging contributor to photoaging through mechanisms including reactive oxygen species (ROS) generation, induction of melanogenesis, persistent hyperpigmentation, and impairment of DNA repair [4].

Photoprotection involves a combination of sun avoidance, use of protective clothing, hats and sunglasses, seeking shade, and appropriate application of sunscreen [5]. However, sunscreen products remain the most effective and widely used approach for protecting the skin from UV-induced damage [6,7]. Sunscreens reduce the harmful effects of UV radiation by absorbing, reflecting, or scattering photons and include physical agents, which are especially important for individuals sensitive to UVA and visible light, and chemical agents, which are more cosmetically appealing and selectively absorb UVB and/or UVA [8]. The American Academy of Dermatology (AAD) and the Canadian Dermatology Association (CDA) recommend using sunscreens with a minimum SPF of 30, which primarily reflects protection against UVB radiation, while broad-spectrum labeling indicates adequate UVA coverage [5]. Protection against visible light is variable and generally limited to specific formulations, such as physical or tinted sunscreens [5]. In addition, physical sunscreens are more difficult to apply evenly than chemical sunscreens, resulting in reduced quantities being used and consequently a lower effective SPF [9]. However, chemical sunscreens can be irritating or poorly tolerated in some individuals [10]. Therefore, most sunscreens combine chemical UV-absorbing filters with physical inorganic sunscreens that reflect UV radiation to provide broad-spectrum protection [10]. Zinc oxide and titanium dioxide are widely used physical sunscreens that have been approved as inorganic compounds protecting against a broad range of UV radiation [5]. On the other hand, the most commonly used chemical sunscreen filters include oxybenzone, avobenzone, octocrylene, octinoxate, homosalate, and octisalate [11]. Although these UV filters provide effective protection, concerns have been raised regarding their photostability, potential skin irritation, environmental persistence, and ecological impact, particularly in marine ecosystems [12]. These concerns have encouraged the cosmetic and dermatological industries to explore alternative formulation strategies that enhance sunscreen efficacy while improving skin tolerability and environmental sustainability.

Natural oils have gained increasing attention as sunscreen ingredients due to consumer demand for natural cosmetics and concerns regarding the safety of organic UV filters [13]. Previous studies have shown that oils including olive, coconut, almond, castor, basil, pomegranate, and shea can contribute to SPF enhancement and allow a reduction in the concentration of organic UV filters while maintaining effective sun protection [14,15,16]. In particular, olive oil and coconut oil demonstrated SPF values of approximately 8, while castor oil and almond oil showed lower SPF values of around 6 and 5, respectively [13,17]. In addition, pomegranate and shea oils have demonstrated antioxidant and photoprotective effects, supporting their potential use as natural UV-filter enhancers in oil-based sunscreen formulations [14,15,16]. Beyond serving as emollients or lipid carriers, many natural oils contain bioactive compounds, including fatty acids, antioxidants, and anti-inflammatory agents, which can help mitigate UV-induced oxidative stress and inflammatory responses [18]. These biological activities are particularly relevant because oxidative stress and inflammation are central mechanisms underlying photoaging and UV-mediated skin damage [19,20]. In this context, natural oils have gained increasing attention as multifunctional cosmetic ingredients, including for UV protection [21,22]. Consequently, incorporating biologically active oils into sunscreen systems may provide complementary protection that extends beyond simple UV filtering, contributing to improved skin health and long-term protection.

Sustainability has also become a major driving force in cosmetic ingredient innovation. Conventional plant-derived oils often require extensive land use, water consumption, and agricultural inputs, which can limit scalability and raise environmental concerns [23,24]. In contrast, insect-derived oils represent a promising and underexplored alternative lipid source. Insects require minimal land and water resources, exhibit high feed conversion efficiency, and generate lower greenhouse gas emissions compared with traditional livestock and many crop-based oil sources [25,26]. As a result, insect-based ingredients align well with global sustainability goals and the increasing demand for environmentally responsible cosmetic products. Among edible insects, crickets have emerged as one of the most suitable candidates for oil production due to their relatively high lipid content, ease of cultivation, and established acceptance in food and feed industries [27,28,29]. Cricket oil exhibits favorable fatty acid composition and physicochemical properties, indicating strong potential as an edible oil ingredient for use in food products [28]. Additionally, cricket oil demonstrates favorable antioxidant activity and oxidative stability, highlighting its strong potential as a high-value oil ingredient for cosmetic applications [29]. Moreover, recent studies have demonstrated that cricket oils from different species can be safely formulated into nanoemulsions, showing no irritation and a suitable hydrophilic–lipophilic balance for topical use [27]. Despite these promising attributes, the application of cricket oil in sunscreen systems has not been investigated. Most previous studies on insect-derived oils have focused on nutritional value, food applications, or basic physicochemical characterization.

Therefore, the present study aimed to develop and evaluate cricket oil-based sunscreen systems with a focus on formulation design, UV protection performance, and preclinical safety. Cricket oils extracted from different species were first characterized for their sun protection factor (SPF), persistent pigment darkening/ultraviolet A protection factor (PPD/UVA-PF), and blue light protection. Sunscreen creams incorporating cricket oil were formulated using both physical and chemical UV filters, and their physicochemical properties were evaluated.

## 2. Materials and Methods

### 2.1. Cricket Materials

Different frozen cricket species, including field crickets (*Gryllus bimaculatus*), ground crickets (*Teleogryllus mitratus*), and house crickets (*Acheta domesticus*), commercially available as edible insects, were obtained from a local farm in Chiang Mai, Thailand. The crickets were dried in a hot air oven (Memmert UM 500, Memmert GmbH & Co., Schwabach, Germany) set at 45 ± 2° C until complete dehydration was achieved. Each dried cricket material was kept in a sealed container at room temperature until further analysis.

### 2.2. Chemical Materials

Avobenzone, Beige EasyMix™ (mica, iron oxides, CI 77491, CI 77492, dimethicone), boron trifluoride (BF_3_), butylene glycol, caprylic/capric triglyceride (Myritol^®^ 318), caprylhydroxamic acid, carbomer^®^ U21 (Acrylates/C10–30 alkyl acrylate crosspolymer), cetyl alcohol (Wax C), cyclomethicone, dimethicone, disodium ethylenediaminetetraacetate (EDTA), glycerin, glyceryl stearate, iron oxides (CI 77491, CI 77492), isononyl isononanoate (LipidSoft™ Lite), lauryl glucoside, lauryl PEG-9 polydimethylsiloxyethyl dimethicone, octisalate, Oil Blender™ (polyglyceryl-3 diisostearate), olive oil, PEG-10 dimethicone, PEG-100 stearate, polyglyceryl-3 diisostearate, titanium dioxide, tocopherol (vitamin e), triethanolamine, xanthan gum, zinc oxide, and 1,2-hexanediol were purchased from Chanjao Longevity Co., Ltd., Bangkok, Thailand. Novemer™ EC-2 (Sodium acrylates/beheneth-25 methacrylate crosspolymer, hydrogenated polydecene, lauryl glucoside) and Spectrastat™ BHL (caprylhydroxamic acid, 1,2-hexanediol, butylene glycol) were purchased from Namsiang Co., Ltd., Bangkok, Thailand. Hexane (≥98.50%) and methanol (≥99.5%) were analytical grade purchased from Merck (Darmstadt, Germany). Crystal violet (≥90%), dimethyl sulfoxide (DMSO; ≥99.9%), fetal bovine serum (FBS; impurities ≤10 EU/mL endotoxin), hydrogen peroxide (H_2_O_2_; 30%), lipopolysaccharide (LPS; <2.5% protein, 5–10% 2-keto-3-deoxyoctonate), N-nitro-L-arginine methyl ester (L-NAME; ≥98%), sodium chloride (≥99.0%), paraformaldehyde (95%), disodium hydrogen phosphate, sodium dihydrogen phosphate, sodium hydroxide, sodium lauryl sulfate, Trolox (6-Hydroxy-2,5,7,8-tetramethylchroman-2-carboxylic acid), and trypsin were analytical grade purchased from Sigma-Aldrich (St. Louis, MO, USA).

### 2.3. Cricket Oil Extraction

Lipid extraction was subsequently performed by mechanical cold pressing using a cold-pressing machine (FEA-100SS-M-H-TC, Energy Friend Ltd. Part., Chiang Mai, Thailand) [30]. The oils obtained from *G. bimaculatus* (GBO), *T. mitratus* (TMO), and *A. domesticus* (ADO) were kept in sealed amber glass containers at ambient temperature until further analysis.

### 2.4. Chemical Composition Analysis of Cricket Oil

Quality control of each cricket oil was performed by analyzing the types and quantities of fatty acids in the extracted cricket oils using a fatty acid methyl ester/gas chromatography–mass spectrometry (FAME/GC–MS) approach according to the method of Prommaban et al. (2021) [31]. Prior to analysis, fatty acid methyl esters were prepared via acid-catalyzed transesterification of the lipid fraction with methanol. Briefly, the oil sample was saponified in 0.5 M methanolic NaOH at 100 °C for 15 min and subsequently derivatized with boron trifluoride (BF_3_) for 1 min after cooling to room temperature. Hexane and a saturated sodium chloride solution were then added, and the hexane layer containing the FAMEs was collected for GC–MS injection. All GC–MS analyses were conducted under Good Manufacturing Practice (GMP), Hazard Analysis and Critical Control Points (HACCP), and Halal Quality and Hygiene System/ISO 22000 Food Safety Management Systems standards [32] at the Research and Service Laboratory, Halal Science Center, Chulalongkorn University, Bangkok, Thailand. Fatty acid types and quantities were identified by comparing retention times with those of standard compounds.

### 2.5. Safety Evaluation of Cricket Oil

#### 2.5.1. Cytotoxicity Assessment of Cricket Oil Using the 3-(4,5-Dimethylthiazol-2-yl)-2,5-diphenyltetrazolium Bromide (MTT) Assay

The cellular safety of cricket oil was evaluated in normal human dermal fibroblasts (NHDF) by measuring mitochondrial activity using the MTT assay following the method of Chomchalao et al. (2024) [33]. In brief, NHDF cells were cultured in DMEM supplemented with 10% fetal bovine serum (FBS) and 1% antibiotics in 96-well plates and incubated at 37 °C with 5% CO_2_ for 24 h or approximately 80% confluence. Cells were then detached using 0.25% trypsin, counted, and seeded at a density of 10,000 cells/well in 96-well plates. After 24 h of incubation, cells were treated with DMEM-F12 containing each cricket oil at concentrations of 25, 50, 100, and 250 µg/mL (100 µL/well). Untreated cells cultured in DMEM-F12 without oil served as the control group. After 24 h of treatment, MTT solution (0.5 mg/mL, 100 µL/well) was added and incubated at 37 °C in the dark for 2 h. The MTT solution was then removed, and 100 µL of DMSO was added to dissolve the formazan crystals. The plate was gently shaken to ensure homogeneity, and absorbance was measured at 570 nm using a microplate reader. Cell viability (%) was calculated using the following equation:

Cell viability (%) = (A/B) × 100,(1)

where A represents the absorbance of treated cells and B represents the absorbance of control cells. All the experiments were conducted in triplicate.

#### 2.5.2. Evaluation of Cell Morphology and Density

NHDF cell morphology and density were examined to further confirm the safety of cricket oil using crystal violet staining. In brief, NHDF cells were seeded at 10,000 cells/well in 96-well plates and treated with cricket oil at various concentrations as described for the MTT assay, with untreated cells as controls. After 24 h, cells were fixed with 4% paraformaldehyde for 1 h and stained with 0.5% crystal violet for 30 min. Excess stain was removed by rinsing with distilled (DI) water, and plates were dried at room temperature. Cell morphology and density were observed and photographed under an inverted microscope (Primo Vert, ZEISS, Carl Zeiss MicroImaging GmbH, Jena, Germany) using brightfield mode.

#### 2.5.3. Hen’s Egg Test on the Chorioallantoic Membrane (HET-CAM) Irritation Test

The irritation potential of cricket oil was assessed using the HET-CAM assay [34]. In brief, fertilized chicken eggs (7–9 days old) were prepared by removing the air sac area using a rotating dentist saw blade, followed by careful removal of the eggshell. Normal saline solution (NSS) was applied to moisten and soften the inner shell membrane, thereby facilitating its gentle removal without damaging the chorioallantoic membrane (CAM). After the application of NSS, the eggs were allowed to stand for approximately 15 min to ensure adequate hydration and loosening of the membrane. The softened inner shell membrane was then carefully removed using forceps, and 30 µL of cricket oil was applied directly onto the CAM. Reactions were observed for 5 min (short-term) and 60 min (long-term). The onset times of vascular hemorrhage, vascular lysis, and vascular coagulation were recorded, and the irritation score (IS) was calculated using the following equation [34]:

IS = [(301 − t(h))/300 × 5] + [(301 − t(l))/300 × 7] + [(301 − t(c))/300 × 9],(2)

where t(h), t(l), and t(c) represent the onset times (in seconds) of hemorrhage, lysis, and coagulation within 5 min (300 s), respectively. Irritation severity was classified as follows: non-irritant (0.0–0.9), slight irritant (1.0–4.9), moderate irritant (5.0–8.9), and severe irritant (9.0–21.0) [34]. In the HET-CAM test, 1% *w*/*v* sodium lauryl sulfate (SLS) solution was used as a positive control, and NSS served as the negative control. All the experiments were conducted in triplicate.

### 2.6. Evaluation of Biological Activities at the Cellular Level

#### 2.6.1. Cellular Antioxidant Activity

The antioxidant activity of cricket oil was evaluated in dermal fibroblasts using the 2′,7′-dichlorodihydrofluorescein diacetate (DCFH-DA) assay [35]. Fibroblast cells were seeded at 10,000 cells/well in black 96-well plates and cultured in DMEM-F12 supplemented with 10% FBS and 1% penicillin/streptomycin at 37 °C with 5% CO_2_ for 24 h. Cells were pretreated with cricket oil at concentrations of 100, 250, and 500 µg/mL for 24 h. Trolox (100 µg/mL) was used as a positive control. After pretreatment, cells were incubated with DCFH-DA in the dark for 30 min, washed three times with PBS, and then exposed to 125 µM hydrogen peroxide (H_2_O_2_) for 15 min to induce oxidative stress. Fluorescence intensity was measured using excitation and emission wavelengths of 485 and 535 nm, respectively, using a microplate reader (CLARIO star, BMG Labtech, DKSH Technology Limited, Bangkok, Thailand). GBO and TMO were tested at 100, 250, and 500 µg/mL, whereas ADO was tested at 100 and 250 µg/mL due to cytotoxicity observed at 500 µg/mL. Olive oil was used as a comparative natural oil control and tested at the same concentrations. All the experiments were conducted in triplicate.

#### 2.6.2. Cellular Anti-Inflammatory Activity

Anti-inflammatory activity was evaluated using RAW 264.7 macrophage cells by measuring nitric oxide (NO^•^) production. Cells were seeded at 50,000 cells/well in 96-well plates and incubated for 24 h in a CO_2_ incubator (Thermo Fisher Scientific, Waltham, MA, USA) set at 37 °C, 5% CO_2_, and 95% humidified air. Cells were pretreated with cricket oil (100, 250, and 500 µg/mL) for 30 min, followed by stimulation with lipopolysaccharide (LPS, 1 µg/mL). After 24 h, culture supernatants were reacted with Griess reagent and incubated at room temperature for 20 min. Absorbance was measured at 540 nm using a CLARIOstar microplate reader (BMG Labtech; distributed by DKSH Technology Limited, Thailand), and NO^•^ concentration was calculated using a sodium nitrite standard curve. Results were compared with LPS-only controls, olive oil-treated groups, and the positive control group treated with N-nitro-L-arginine methyl ester (L-NAME). All oils were confirmed to be non-toxic to macrophages at the tested concentrations by MTT assay.

### 2.7. Sun Protection Factor (SPF) Evaluation of Cricket Oil

The sun protection ability of cricket oil was evaluated using in vitro spectrophotometric methods based on absorbance measurements. For SPF determination, the absorbance of a dilute hydroalcoholic solution of the sample (0.1% *w*/*v*) was measured over the wavelength range of 290–320 nm at 5 nm intervals using a UV–visible spectrophotometer with a 1 cm quartz cuvette at MySkinRecipes Analytical Laboratory, Bangkok, Thailand. The SPF values representing UVB protection were subsequently calculated using the Mansur equation [36]. UVA protection was evaluated using the Persistent Pigment Darkening (PPD/UVA-PF) method over the range of 320–400 nm [37,38]. Blue light protection was calculated as the percentage reduction in light transmission over the wavelength range of 380–450 nm by converting absorbance values to transmittance [39].

### 2.8. Development of Cricket Oil-Based Sunscreen Products

Cricket oil with optimal sun protection, antioxidants, and anti-inflammatory properties was selected for sunscreen formulation.

#### 2.8.1. Development of Base Cream Emulsions

Oil-in-water (O/W) emulsions were prepared using cold processes (Table 1) and hot processes (Table 2). In the cold process, all ingredients were combined at ambient temperature and mixed under continuous stirring until a homogeneous emulsion was obtained. In contrast, in the hot process, the oil and aqueous phases were heated separately, with the oil phase heated to 70 °C and the aqueous phase to 75 °C. The aqueous phase was then gradually added to the oil phase under continuous stirring until cooling to room temperature to yield a stable emulsion. Formulations were screened for homogeneity, texture, and phase stability prior to the incorporation of UV filters. Homogeneity was evaluated by visual inspection for uniformity and absence of phase separation. Texture was assessed manually based on spreadability and consistency when applied to a watch glass. Phase stability was examined by centrifugation and further assessed through accelerated temperature cycling between 45 °C and 4 °C for six cycles, with subsequent visual inspection.

#### 2.8.2. Development of Cricket Oil Sunscreen Formulations

Selected base formulations were supplemented with both physical and chemical ultraviolet (UV) filters. Briefly, physical UV filters (titanium dioxide and zinc oxide), chemical UV filters (avobenzone and oxybenzone), as well as emulsifiers, preservatives, humectants, emollients, stabilizers, and fragrances were incorporated into the formulations. The composition and functional roles of each ingredient are summarized in Table 3. Emulsions were prepared by separately heating the aqueous and oil phases, followed by homogenization and the stepwise incorporation of active ingredients. The physicochemical properties of the sunscreen formulations, including physical appearance, viscosity (determined using a Brookfield DVIII rheometer, Brookfield Engineering Laboratories, Stoughton, MA, USA), and pH (determined using ColorpHast^®^ 9590-3 pH test strips, pH 0–14, Merck KGaA, Darmstadt, Germany), were evaluated. Formulation stability was assessed using centrifugation and accelerated temperature cycling between 45 °C and 4 °C for six cycles, followed by visual inspection for phase separation or physical changes. The SPF, UVA protection (PPD/UVA-PF), and blue light protection of the cricket oil-based sunscreen formulations were determined using spectrophotometric methods and compared with olive oil-based sunscreen formulations commonly employed in cosmetic applications.

To evaluate the superior effect of cricket oil compared to olive oil, the most suitable sunscreen formulation, which provided the highest SPF, PPD/UVA-PF, and blue light protection, was selected for formulations containing either cricket oil or olive oil. The formulations were prepared using TMO or olive oil as the base, with or without the incorporation of sunscreen agents. The formulations were coded and are summarized in Table 4. Blank denotes the formulation without any oil or UV filters, COB denotes the TMO-based cream formulation without UV filters, COS denotes the TMO-based sunscreen cream formulation, OOB denotes the olive oil-based cream formulation without UV filters, and OOS denotes the olive oil-based sunscreen cream formulation. Additionally, the effect of pigment incorporation was also assessed, as summarized in the formulations listed in Table 4. Formulations containing pigments were designated by the addition of the letter “P” to the corresponding code. COBP and COSP represent the TMO-based cream and sunscreen formulations with pigments, respectively, whereas OOBP and OOSP denote the corresponding olive oil-based formulations containing pigments.

### 2.9. Statistical Analysis

All data are expressed as mean ± standard deviation. Statistical analyses were performed using GraphPad Prism v10.2.3 (GraphPad Software Inc., San Diego, CA, USA). Differences among multiple groups were evaluated by one-way analysis of variance (ANOVA) followed by Tukey’s post hoc test, while comparisons between two groups were conducted using a paired *t*-test. A *p*-value of less than 0.05 was considered statistically significant. Statistical differences among groups are indicated by different lowercase letters (a–f), where values sharing the same letter are not significantly different, and values with different letters indicate statistically significant differences.

## 3. Results and Discussion

### 3.1. Cricket Oils and Chemical Compositions

**Figure 1:**
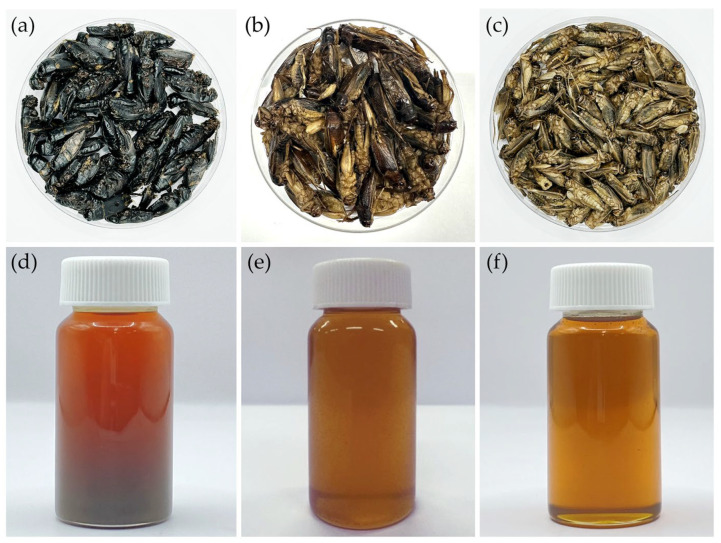
Physical characteristics of different types of dried crickets, including *G. bimaculatus* (**a**), *T. mitratus* (**b**), and *A. domesticus* (**c**), along with cold-pressed oils extracted from *G. bimaculatus* (**d**), *T. mitratus* (**e**), and *A. domesticus* (**f**).

The physical appearances of dried crickets and their corresponding cold-pressed oils are shown in Figure 1. Noticeable differences were observed among species. Dried *G. bimaculatus* exhibited a darker coloration compared with *T. mitratus* and *A. domesticus*. The varied coloration observed among different cricket species may reflect species-specific differences in cuticle pigmentation and sclerotization processes, particularly in the relative allocation of dopamine toward melanin versus yellow sclerotin synthesis [40]. In *G. bimaculatus*, the dark body coloration is likely explained by reduced or disrupted expression of the ebony gene, which impairs the conversion of dopamine into N-β-alanyl dopamine, the precursor of yellow sclerotin [40,41]. Consequently, excess dopamine is redirected toward melanin synthesis, leading to increased melanin deposition and cuticle darkening during development [42].

Similar to dried crickets, extracted cricket oils also varied in color among species. GBO appeared darker reddish-brown, whereas TMO and ADO were lighter yellow to amber, consistent with the coloration observed in the corresponding dried cricket materials. These differences may be associated with variations in pigment content and the presence of minor compounds co-extracted during cold pressing [29]. Previous studies have reported that the darker color of screw-pressed cricket oil results from the co-extraction of polar compounds, phospholipids, oxidation products, and darker pigments in addition to non-lipid components [29]. Furthermore, the yellowness of cricket oil has been attributed to the presence of carotenoid pigments, including lutein, canthaxanthin, β-cryptoxanthin, and β-carotene [29].

The yields of dried crickets obtained from hot-air drying are presented in Table 5 and compared with those obtained from freeze-drying in our previous study [27]. For hot-air drying, yields ranged from 28.7 to 30.2% (*w*/*w*, based on fresh weight), with *G. bimaculatus* exhibiting the highest yield, followed by *T. mitratus* and *A. domesticus*. A similar species-dependent trend was observed for freeze-drying but with slightly lower yields, ranging from 27.6 to 28.9% [27]. Although freeze-drying is a dehydration process that removes ice from frozen material by sublimation at low temperatures, thereby minimizing deterioration and microbial activity, preventing shrinkage, and preserving overall product quality [43,44], hot-air drying, which removes moisture by convective heat transfer and is often associated with greater structural and quality changes, is widely used [45]. The current study showed that both drying methods had comparable effects on the yield of crickets. Additionally, a previous study reported that oven-drying and freeze-drying of blanched *Ruspolia differens* resulted in comparable composition and nutritional quality, including proximate, mineral, and fatty acid profiles [46]. The comparable effects of freeze-drying and hot-air drying in insects are likely due to their rigid exoskeleton, as opposed to fruits and vegetables, where freeze-drying is superior because it minimizes shrinkage and better preserves shape and structure.

Additionally, the yields of cricket oils obtained from hot-air drying are presented in Table 5 for comparison with those obtained from freeze-drying in our previous study [27]. The pattern of cricket oil yield observed in the present study was consistent with our previous findings [27,47], in which *G. bimaculatus* produced the highest oil yield, followed by *T. mitratus* and *A. domesticus*. In addition, the findings were in good agreement with the literature, which reports that *G. bimaculatus* contained a high lipid content ranging from 11.90 to 20.74% *w*/*w*, which is higher than that of *A. domesticus*, with a reported lipid yield of approximately 12.2% *w*/*w* [48]. These results indicate that cricket species significantly influence oil yield, as each species possesses distinct biological characteristics, particularly with respect to lipid accumulation within the body [49].

On the other hand, cricket oil yields did not differ markedly between the drying methods. Hot-air-dried crickets showed oil yields of 13.7–17.9% on a dry-weight basis (3.9–5.4% fresh weight), while freeze-dried samples yielded 15.5–24.6% (4.3–6.8% fresh weight), with *G. bimaculatus* showing the highest values in both cases. Although differences were apparent when yields were compared on a dry-weight basis, calculation to a fresh-weight basis revealed only minor differences, indicating that hot-air drying and freeze-drying provide comparable oil recovery and can be used interchangeably depending on cost and practical considerations. Although freeze-drying provided slightly higher oil yields, its higher processing cost must be considered for large-scale production, making hot-air drying a more practical alternative.

Nowadays, insect-derived oils are increasingly recognized as promising alternatives to conventional lipid sources due to their high lipid yields, lower environmental impact, and potential for production using low-cost waste feedstocks [49]. Additionally, replacing vegetable oil with insect oil in food products is nowadays increasingly attractive because insect oils offer high lipid yields and improved sustainability while maintaining sensory quality [50]. Therefore, insect oils show strong potential for cosmetic applications. The findings of this study reveal that among the different cricket species, *G. bimaculatus* consistently exhibited the highest drying and oil yields regardless of the drying method, suggesting its suitability for industrial applications targeting both dried cricket products and cricket oil extraction.

The fatty acid compositions of oils extracted from different cricket species were analyzed and compared with olive oil, a widely used reference oil in cosmetic and dermatological applications due to its well-documented emollient properties, skin compatibility, and photoprotective potential [17]. The results are presented in Table 6. Cricket oils from all species exhibited substantially higher levels of saturated fatty acids (SFAs) compared with olive oil. Palmitic acid (C16:0) was the predominant SFA in cricket oils, ranging from 25.81 to 26.25%, which was approximately double that found in olive oil (12.94%). Stearic acid (C18:0) was also present at higher concentrations in cricket oils (6.73–9.18%) than in olive oil (3.37%). In addition, several medium- and long-chain SFAs, including myristic acid (C14:0), lauric acid (C12:0), margaric acid (C17:0), pentadecanoic acid (C15:0), and behenic acid (C22:0), were detected in cricket oils but were absent or present only in trace amounts in olive oil. These SFAs may enhance the structural stability and oxidative resistance of cricket oils while contributing to skin barrier function by improving turgor, firmness, smoothness, and softness and reducing transepidermal water loss through an occlusive protective effect, which is advantageous for topical formulations [51].

In contrast, olive oil was characterized by a markedly higher proportion of monounsaturated fatty acids (MUFAs), dominated by oleic acid (C18:1), which accounted for 73.92% of total fatty acids. Although oleic acid was also the major MUFA in cricket oils, its content was considerably lower, ranging from 29.90 to 31.70%. Minor MUFAs such as palmitoleic acid (C16:1), myristoleic acid (C14:1), cis-11-eicosenoic acid (C20:1), and erucic acid (C22:1) were detected in cricket oils in small quantities, further highlighting species-specific differences in lipid composition. Notably, cricket oils contained significantly higher levels of polyunsaturated fatty acids (PUFAs) than olive oil. Linoleic acid (C18:2) was particularly abundant in cricket oils, reaching up to 31.27% in ADO, compared with only 7.21% in olive oil. Linolenic acid (C18:3) was also present at higher levels in cricket oils (0.77–1.15%) than in olive oil (0.66%). Furthermore, certain long-chain PUFAs, including arachidonic acid (C20:4) and eicosatrienoic acids (C20:3), were detected exclusively in cricket oils. These PUFAs are known to play important roles in improving skin barrier function, inhibiting UV-induced inflammation and hyperpigmentation, attenuating dry skin and pruritus associated with dermatitis, and accelerating wound healing, thereby highlighting their strong potential for cosmetic and cosmeceutical applications [52]. Comparison with olive oil provides a meaningful benchmark because it is well documented in cosmetic and dermatological research for its high oleic acid content, emollient properties, and bioactive phenolics that provide antioxidant, anti-inflammatory, photoprotective, wound-healing, and skin-barrier benefits [53,54]. While cricket oils differ substantially from olive oil in fatty acid composition, particularly in their higher PUFA and SFA contents, these differences may offer complementary or alternative functional properties. The elevated PUFA content in cricket oils may enhance skin hydration and barrier repair [55], whereas the presence of diverse saturated fatty acids may improve formulation stability. The unique lipid composition highlights the potential of cricket oil as a novel and sustainable ingredient for cosmetics, either as a partial substitute for or in combination with conventional plant-based oils.

### 3.2. Safety of Cricket Oil

**Figure 2:**
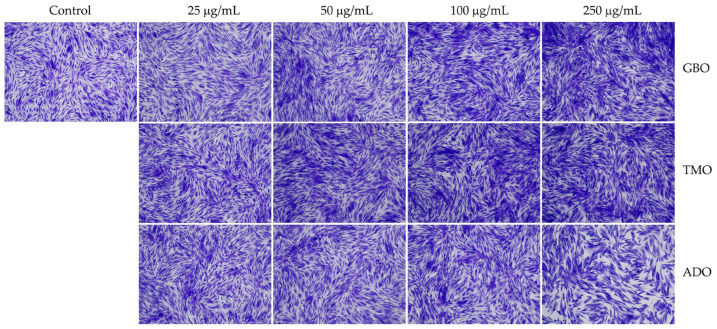
Morphological characteristics of NHDF fibroblast cells following crystal violet staining with no treatment (Control) and after 24 h of exposure to cricket oil, including *Gryllus bimaculatus* (GBO), *Teleogryllus mitratus* (TMO), and *Acheta domesticus* (ADO).

**Figure 3:**
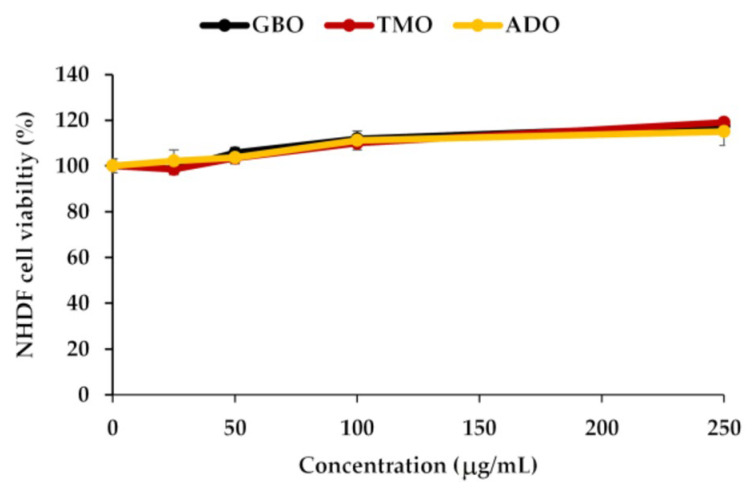
Viable NHDF fibroblast cells after exposure to different cricket oils, including *Gryllus bimaculatus* (GBO), *Teleogryllus mitratus* (TMO), and *Acheta domesticus* (ADO).

The safety of cricket oil was evaluated using cytotoxicity testing in NHDF cells (Figure 2 and Figure 3) and the HET-CAM test (Table 7). Cytotoxicity testing in NHDF fibroblast cells was performed to evaluate the direct effects of cricket oil on human skin cells, as fibroblasts play a crucial role in maintaining skin structure, wound healing, and extracellular matrix production [56,57,58]. Assessing cell viability after exposure allows determination of whether cricket oil induces cellular damage, inhibits cell growth, or causes cell death, thereby indicating its biocompatibility and safety for topical or cosmetic use [59,60]. Both morphological observation and quantitative cell viability are shown in Figure 2 and Figure 3, respectively. The microscopic images illustrate the morphology of NHDF fibroblast cells after treatment with different concentrations of cricket oils (GBO, TMO, and ADO) compared with the untreated control. In the control group, NHDF cells exhibited a typical fibroblast morphology characterized by an elongated, spindle-shaped structure and uniform cell distribution [61]. Similarly, cells treated with all cricket oils at every tested concentration maintained comparable morphological characteristics. The cells showed no evidence of cell shrinkage or death. As the concentration of cricket oil increased, no morphological deterioration of NHDF fibroblast cells was observed. Instead, a slight increase in cell density was evident at higher concentrations of 100 and 250 µg/mL, which correlated well with the elevated cell viability values shown in Figure 3. All cricket oils demonstrated excellent cytocompatibility across the tested concentration range (25–250 µg/mL), with cell viability consistently remaining at or above 100% relative to the initial cell numbers. Furthermore, a clear concentration-dependent enhancement in cell viability was observed for all oils, suggesting a potential stimulatory effect on cellular metabolic activity or proliferation, with the highest viability values ranging from 115.0 ± 6.1% to 119.1 ± 1.7% at 250 µg/mL. This concentration-dependent increase in cell viability indicates that the cricket oils did not induce cytotoxic effects on NHDF fibroblast cells and may instead promote cellular metabolic activity or proliferation. The maintenance of normal fibroblast morphology, together with enhanced cell viability at higher concentrations, suggests that the oils support cell growth rather than causing cellular stress or damage. The current findings demonstrated the safety of cricket oils for contact with human skin cells and support their potential application in cosmetic or dermatological formulations.

The HET-CAM assay was employed to evaluate the irritation potential of cricket oil on vascularized tissue. The CAM is highly sensitive and richly vascularized, making it an appropriate model for detecting irritation responses such as hemorrhage, vascular lysis, and coagulation [62]. This assay is widely recognized as an alternative to animal testing, providing a rapid and reliable assessment of irritation potential while adhering to ethical guidelines [63]. When combined with the in vitro cytotoxicity assay, the HET-CAM test enables a comprehensive evaluation of cricket oil safety at both the cellular and tissue levels. Hemorrhage, vascular lysis, and coagulation are macroscopic endpoints directly observed on the CAM following exposure to the test substance under a stereomicroscope. Hemorrhage refers to the leakage of blood from damaged blood vessels, observed as blood spreading into the surrounding membrane [64]. Vascular lysis occurs when the integrity of the vascular cell membranes is disrupted, causing the vessels to break down and cellular components to be released [64]. Coagulation is the condition in which blood solidifies or clots within the vessels, leading to vessel blockage and cessation of blood flow [64]. As shown in Table 7, the positive control induced pronounced vascular damage, including hemorrhage, vascular lysis, and coagulation, which became evident within 5-min observation period and intensified over time. A high irritation score of 18.5 ± 0.1 was observed for the positive control, classifying it as a severe irritant [65], whereas the negative control showed no observable vascular changes throughout the entire observation period. In addition, all cricket oil samples induced no signs of hemorrhage, vascular lysis, or coagulation, resulting in irritation scores of 0.0 ± 0.0 and classification as non-irritating. These results suggest that cricket oils are safely tolerated and do not adversely affect vascular integrity. When considered together with the cytotoxicity results, the HET-CAM outcomes further support the safety and biocompatibility of cricket oils for further topical applications.

### 3.3. Antioxidant and Anti-Inflammatory Activities of Cricket Oils

**Figure 4:**
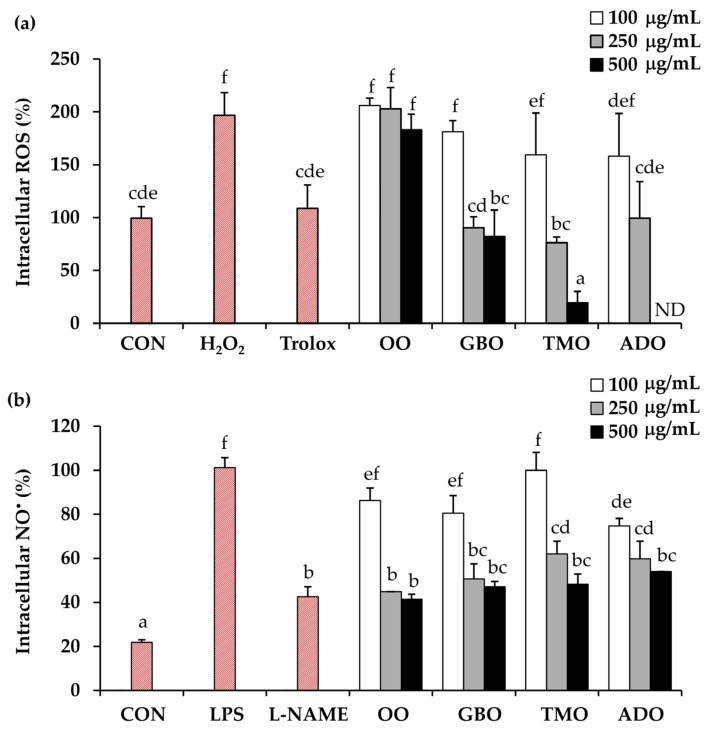
Intracellular reactive oxygen species (ROS) (**a**) and nitric oxide (NO^•^) levels (**b**) following stimulation with hydrogen peroxide (H_2_O_2_) and lipopolysaccharide (LPS), respectively, and after treatment with Trolox, Nω-nitro-L-arginine methyl ester (L-NAME), olive oil (OO), and different cricket oils, including *Gryllus bimaculatus* (GBO), *Teleogryllus mitratus* (TMO), and *Acheta domesticus* (ADO), compared with a control group (CON) receiving no treatment. The red bars represent the control group (CON), negative control, and positive control in each assay. Different letters (a–f) denote statistically significant differences among groups as determined by one-way ANOVA followed by Tukey’s post hoc test (*p* < 0.05).

The intracellular ROS and NO^•^ levels following treatment with the cricket oils, in comparison with olive oil, are shown in Figure 4. Hydrogen peroxide (H_2_O_2_) was used as an oxidative stress-inducing agent by directly damaging cellular components or by generating highly reactive secondary radicals, such as hydroxyl radicals, which further contribute to inflammation and cellular aging [66,67]. Therefore, the ROS levels doubled after the H_2_O_2_ treatment when compared with the control. Trolox, used as a reference antioxidant, significantly reduced intracellular ROS levels, thereby validating the assay conditions. Olive oil exhibited consistently high ROS levels across all tested concentrations, indicating limited antioxidant activity. In contrast, all cricket oils demonstrated a concentration-dependent reduction in intracellular ROS. Among the tested samples, TMO exhibited the most pronounced ROS-scavenging activity at higher concentrations, particularly at 500 µg/mL, where intracellular ROS levels were significantly lower than those observed in the oil-treated samples. Notably, ROS levels in the TMO-treated group were reduced to levels lower than those of the untreated control and Trolox-treated cells, suggesting a strong ROS-scavenging capacity rather than a pro-oxidant effect. ADO also reduced ROS production; however, its effect at high concentration (500 µg/mL) could not be evaluated due to cytotoxicity. TMO was found to be the most attractive oil for reducing intracellular ROS levels. As excessive production of ROS is a major contributor to oxidative stress, which leads to cellular damage, inflammation, and skin aging through oxidation of lipids, proteins, and DNA [68,69], reduction in ROS generation or effective scavenging of ROS helps maintain redox homeostasis, protects cellular structures, and prevents the activation of oxidative stress-related inflammatory pathways [70,71]. Therefore, agents capable of reducing intracellular ROS levels play an important role in preserving skin integrity and cellular function.

In addition to their antioxidant effects, the anti-inflammatory potential of the cricket oils was evaluated by measuring intracellular nitric oxide (NO^•^) production following LPS stimulation. The results showed that LPS markedly increased intracellular NO^•^ levels, thereby confirming the validity of the assay. LPS is known to induce inducible nitric oxide synthase (iNOS) in various cell types, including macrophages, vascular smooth muscle cells, and endothelial cells, leading to excessive NO^•^ production [72]. At elevated concentrations, NO^•^ is rapidly converted into reactive nitrogen species (RNS) that modify signaling proteins, impair mitochondrial respiration, and amplify inflammatory and cytotoxic processes [73,74,75]. Consequently, excessive NO^•^ production is closely associated with inflammatory tissue damage [72,73,74]. Therefore, reducing or inhibiting excessive NO^•^ generation is generally considered beneficial, as it can attenuate inflammatory responses and limit cellular injury. However, because NO^•^ also plays important physiological roles in host defense and cellular signaling, complete suppression of NO^•^ is not desirable [73]. Instead, modulation of pathological NO^•^ overproduction is viewed as a favorable anti-inflammatory strategy. L-NAME was used as a reference inhibitor in the current study because it is a well-established nitric oxide synthase (NOS) inhibitor that effectively suppresses NO^•^ production by blocking the conversion of L-arginine to NO^•^ [76,77]. Its use therefore serves as a positive control for NO^•^ inhibition in inflammation-related assays.

In addition, natural oils showed potential to inhibit NO^•^ production in a concentration-dependent manner (Figure 4b). At a low concentration of 100 μg/mL, most oils had no significant effect on NO^•^ levels compared with the LPS group, indicating limited inhibitory activity at this dose. However, at higher concentrations, marked inhibition of NO^•^ production was observed, comparable to that of L-NAME. The observed inhibitory effects suggested that these oils may exert anti-inflammatory activity by suppressing inducible nitric oxide synthase (iNOS)-mediated NO^•^ generation. This aligns with previous reports indicating that bioactive compounds in natural oils, such as phenolics and fatty acid derivatives, can interfere with inflammatory signaling pathways [78,79].

### 3.4. SPF of Cricket Oils

SPF, PPD/UVA-PF, and blue light protection of cricket oils are shown in Table 8. Among all oils tested, only TMO exhibited measurable SPF, PPD/UVA-PF, and blue light protection potential. The SPF value was very low (0.04), indicating negligible protection against UVB radiation. In contrast, the PPD/UVA-PF value was 12.1, corresponding to a PA++++ rating, which indicates strong protection against UVA radiation. Additionally, the blue light protection was 1.2%, suggesting minimal effectiveness in shielding against blue light exposure. The findings noted that TMO demonstrates promising potential as a UVA-protective and photoaging-preventive ingredient, rather than as a standalone sunscreen. Its strong UVA protection suggests suitability for use as a functional cosmetic ingredient in formulations aimed at mitigating chronic UV exposure, while requiring combination with conventional UVB and blue light filters for broad-spectrum protection.

### 3.5. Cricket Oil-Based Sunscreen Products

In the development of a sunscreen product from cricket oil, TMO was selected due to its outstanding biological activities, including strong antioxidant and anti-inflammatory effects at the cellular level. It effectively reduces reactive oxygen species (ROS) in fibroblast cells and inhibits nitric oxide (NO^•^) production in macrophages (Figure 4). In addition, it demonstrates high efficacy in UVA protection (PPD/UVA-PF = 12.1, PA+++), which helps delay premature aging and reduce skin damage (Table 8). Furthermore, the TMO exhibited cellular-level safety and did not cause irritation in the HET-CAM assay. Therefore, TMO is considered a suitable ingredient for the development of sunscreen formulations. In this study, a sunscreen cream was formulated as an O/W emulsion, offering favorable rheological and sensorial properties for topical use, including a lightweight texture, high spreadability, and a non-greasy finish [80].

Two emulsification methods were employed, including cold process and hot process emulsification. For the cold process method, TMO was incorporated at varying concentrations ranging from 1–5% *w*/*w*, in combination with Novemer™ EC-2, a pre-neutralized liquid polymer that functions as an emulsifying agent in cream formulations. The physical characteristics of the cold process cream formulations are shown in Table 9. It was found that Novemer™ EC-2 at a concentration of 2% *w*/*w* was insufficient to form a stable emulsion, as the prepared emulsion was unstable after high-speed centrifugation. In contrast, Novemer™ EC-2 at 4% *w*/*w* provided suitable emulsification, resulting in a cream with good stability. The amount of TMO in the formulation significantly affected viscosity. As the concentration increased from 1% *w*/*w* to 5% *w*/*w*, viscosity increased from 1.04 ± 0.1 Pa·s to 3.83 ± 0.1 Pa·s, respectively. However, formulations containing 3% *w*/*w* or more TMO exhibited a homogeneous, pleasant texture and were able to disperse pigments uniformly. Therefore, for cold process cream formulations, it is recommended to use 4% *w*/*w* Novemer™ EC-2 in combination with at least 3% *w*/*w* TMO.

For the hot process emulsification, TMO was incorporated at varying concentrations ranging from 1–5% *w*/*w*, in combination with Wax C (cetyl alcohol), a fatty alcohol that enhances emulsion stability, contributes to cream structure, and increases formulation viscosity. The concentration of Wax C was adjusted according to the TMO content, maintaining a TMO-to-Wax C ratio of 2:1. In addition, other oils were included, particularly those recognized for their moisturizing and skin-softening properties, which impart a soft skin feel and a lightweight formulation texture. These included Myritol^®^ 318 (caprylic/capric triglyceride) and LipidSoft™ Lite (isononyl isononanoate). Emulsifying agents used in the formulation were Lexemul^®^ 561 (glyceryl stearate and PEG-100 stearate), along with Oil Blender (polyglyceryl-3 diisostearate) to improve the dispersion of oil-phase components. For the aqueous phase, xanthan gum and Carbomer U21 were employed as viscosity-enhancing agents, which effectively contributed to improved emulsion stability. The physical characteristics of the cream formulations prepared using the hot process are presented in Table 10.

The results showed that all formulations exhibited good stability, with a pH value of 5.5 ± 0.0, which falls within the range suitable for topical application on human skin [81]. The pH remained unchanged with increasing oil content, indicating that adjustments in the formulation components did not affect the acid–base balance of the system. Differences in viscosity among formulations were observed and were clearly influenced by the proportions of TMO and Wax C. As the concentration of these components increased, the viscosity of the cream tended to increase, reflecting the role of both substances in reinforcing the structure of the emulsion system. Statistical analysis revealed that formulations containing 3% *w*/*w* TMO were able to accommodate a higher oil content without a statistically significant increase in viscosity (*p* > 0.05) when compared with formulations containing a lower oil concentration (2% *w*/*w*). In contrast, formulations with higher oil content (4% *w*/*w*) exhibited a statistically significant increase in viscosity, which could negatively impact spreadability and ease of application. In addition to viscosity, the 3% *w*/*w* formulation maintained desirable texture, smoothness, and spreadability during physical evaluation. Therefore, the formulation containing 3% *w*/*w* TMO was considered the most appropriate, as it provided an optimal balance between increasing the amount of cricket oil and maintaining cream viscosity at a level suitable for practical use.

A comparison between sunscreen cream formulations prepared using the cold process and the hot process revealed that both methods produced formulations with good stability and pH values within the skin-compatible range [81]. However, when physical properties and application suitability were considered together, formulations prepared using the hot process were found to be more appropriate. In the cold process formulations, although the use of 4% *w*/*w* Novemer™ EC-2 successfully produced stable emulsions, the viscosity increased significantly with each incremental increase in TMO concentration (*p* < 0.05), potentially resulting in an excessively thick texture at higher oil levels. In contrast, formulations prepared using the hot process, incorporating Wax C in combination with an emulsifier system and viscosity-enhancing agents, demonstrated a greater capacity to accommodate increased TMO content. In particular, formulations containing 3% *w*/*w* TMO showed no statistically significant difference in viscosity compared with formulations containing 2% *w*/*w* oil (*p* > 0.05), whereas formulations containing 4% *w*/*w* or more began to exhibit a significant increase in viscosity. Consequently, based on the comparative evaluation of both preparation methods, the hot process sunscreen cream formulation containing 3% *w*/*w* TMO was selected as the optimal formulation. This formulation allows for the incorporation of a sufficient level of biologically active TMO without excessively increasing viscosity, resulting in a cream that achieves a balanced combination of stability, texture, and suitability for topical application.

In the development of a sunscreen formulation incorporating TMO, which exhibits antioxidant and anti-inflammatory activities at the cellular level, as well as high efficacy in UVA protection (PPD/UVA-PF = 12.1, PA+++), the oil was shown to help delay premature skin aging, reduce skin damage, and provide moderate protection against blue light exposure. In addition, TMO demonstrates cellular-level safety and does not induce irritation in the HET-CAM assay. However, despite its notable biological activities related to antioxidation and UVA protection, TMO exhibits a low SPF, which reflects limited efficacy against UVB radiation [22]. Therefore, it is insufficient to function as the sole active sunscreen agent in product development. To achieve comprehensive protection against ultraviolet radiation, it is necessary to incorporate physical and/or chemical sunscreen agents into the formulation to enhance UVB protection and increase the SPF to meet regulatory standards. In this context, TMO served as a functional booster ingredient, contributing to the reduction in oxidative stress and inflammation, while physical and chemical sunscreens act as the primary UV-protective agents, thereby providing a more complete skin protection profile.

The physical sunscreen agents used in this study were zinc oxide and titanium dioxide, which function by reflecting and scattering ultraviolet radiation away from the skin [82,83]. Zinc oxide provides broad-spectrum protection, covering both UVA and UVB ranges, whereas titanium dioxide is particularly effective against UVB radiation and short-wavelength UVA [84,85,86]. Consequently, combining these two inorganic filters allows for complementary photoprotection, resulting in enhanced coverage across the entire ultraviolet spectrum [86]. The sunscreen formulations developed with physical sunscreen agents are presented in Table 11, employing different ratios of zinc oxide to titanium dioxide. All formulations demonstrated good physical stability and maintained a pH of 5.5 ± 0.0, which lies within the skin-compatible range. These results indicate that variations in the proportions of the two physical sunscreen agents did not affect the acid–base balance or overall stability of the formulations. In terms of viscosity, formulations with titanium dioxide alone exhibited the highest viscosity (1.40 ± 0.01 Pa·s), whereas zinc oxide alone showed a lower viscosity (0.90 ± 0.01 Pa·s). Upon partial replacement of titanium dioxide with zinc oxide, viscosity decreased markedly. The formulation with a zinc oxide and titanium dioxide ratio of 3:6 and 6:3 showed significantly lower and comparable viscosities.

Regarding UVB and blue light protection, zinc oxide exhibited a more prominent role than titanium dioxide alone. Formulations containing zinc oxide alone achieved an SPF of 16.27 and provided 25.1% blue light protection, whereas formulations containing titanium dioxide alone showed a very low SPF of 0.22 and no measurable blue light protection. The present results differ from previous reports, suggesting that titanium dioxide predominantly filters UVB radiation, while zinc oxide is more effective in the UVA range [87]. The observed differences are likely attributable to variations in particle size and dispersion, with more uniformly dispersed zinc oxide particles enhancing UVB attenuation, while less optimal titanium dioxide dispersion limited its UVB filtering efficiency when used alone [83,87]. Nevertheless, when both physical sunscreen agents were combined, the results were consistent with previous reports, with higher titanium dioxide content contributing primarily to enhanced UVB protection. Additionally, the combined use of both agents resulted in a substantial enhancement of UVB and blue light protection. Formulations containing zinc oxide and titanium dioxide at ratios of 3:6 and 6:3 yielded SPF values of 33.41 and 29.41, respectively, which were significantly higher than those obtained using either agent alone. Additionally, blue light protection increased to 33.8% and 33.0%, respectively, exceeding the protection levels achieved by individual sunscreen agents.

Generally, blue light protection is governed by light scattering in the visible range [88], where zinc oxide is known to perform better than titanium dioxide due to its broader scattering profile. This explains why formulations of zinc oxide exhibited measurable blue light protection, whereas titanium dioxide alone did not. With respect to UVA protection, both zinc oxide and titanium dioxide demonstrated effective performance, with PPD/UVA-PF values of 18.90 and 12.00, respectively. The superior UVA protective performance of zinc oxide is in agreement with previous reports demonstrating that titanium dioxide is more effective against UVB radiation, while zinc oxide exhibits higher efficacy in the UVA range [87]. The combined use of both agents further enhanced UVA protection. Among the evaluated ratios, the zinc oxide to titanium dioxide ratio of 3:6 was identified as the most optimal, as it provided balanced and effective protection against UVA, UVB, and blue light, while maintaining favorable physical properties and suitability for topical application. Consequently, this formulation exhibits strong potential for further development into a commercial sunscreen cream product.

In addition, sunscreen formulations based on cricket oil were developed using chemical sunscreen agents, including octisalate and avobenzone. Octisalate plays a key role in enhancing the SPF and improving the photostability of avobenzone, while avobenzone provides protection against long-wavelength UVA radiation, which is closely associated with premature skin aging [89]. The combined use of chemical sunscreen agents therefore enables more comprehensive and effective ultraviolet protection, particularly when used in conjunction with physical sunscreen agents within the same formulation. The TMO-based sunscreen cream formulations developed with chemical sunscreen agents are presented in Table 12, employing different ratios of octisalate and avobenzone. All formulations exhibited a uniform cream appearance and good physical stability, with a consistent pH of 5.5 ± 0.0, which is suitable for topical application [81]. However, variations in viscosity were observed among the formulations. Avobenzone alone exhibited the highest viscosity (0.97 ± 0.02 Pa.s), whereas octisalate alone showed a comparatively lower viscosity (0.60 ± 0.01 Pa.s). The combination of both chemical sunscreens resulted in a noticeable reduction in viscosity. In terms of photoprotective efficacy, both SPF and PPD/UVA-PF values increased with the total concentration of sunscreen agents. The formulation containing octisalate and avobenzone at a 5:3 ratio demonstrated the highest SPF (40.51) and PPD/UVA-PF (39.17), achieving a PA++++ rating, indicative of effective protection against both UVB and UVA radiation. In addition, blue light protection tended to increase with the appropriate combination of octisalate and avobenzone, with the 5:3 formulation providing the highest level of protection (34.0%). These findings indicate that adjusting the ratio of octisalate to avobenzone not only influences the physical properties of the formulation but also plays a critical role in determining photoprotective performance against UV radiation and high-energy visible light. Formulations incorporating these two chemical sunscreen agents at optimized ratios provide the most favorable balance between stability, sensory properties, and sunscreen efficacy.

To achieve comprehensive protection against UVA, UVB, and blue light, both physical and chemical sunscreen agents were incorporated into the formulations, including 3% *w*/*w* zinc oxide, 6% *w*/*w* titanium dioxide, 5% *w*/*w* octisalate, and 3% *w*/*w* avobenzone. A comparative evaluation between TMO and olive oil-based formulations was conducted, as presented in Table 13. The results clearly demonstrated that TMO exhibited superior sunscreen-enhancing performance. The sunscreen cream formulated with TMO achieved an SPF of 40.20 and a PPD/UVA-PF of 22.83, both of which were higher than those of the olive oil-based sunscreen cream, which showed an SPF of 36.36 and a PPD/UVA-PF of 21.70. In addition, blue light protection was also greater in the TMO formulation compared with the olive oil formulation. In contrast, formulations without sunscreen agents exhibited very low SPF values and minimal blue light protection, indicating that oils alone do not provide effective photoprotection. These findings suggest that while oils themselves do not act as UV-protective agents, TMO can function as an effective performance-enhancing base oil when combined with sunscreen filters, improving the overall photoprotective efficacy of the formulation more effectively than olive oil. All formulations maintained appropriate pH values and good physical stability, highlighting the strong potential of TMO as a novel alternative base oil for sunscreen and skin-protective cosmetic products.

In addition, foundation pigments were incorporated into the formulations to enhance their suitability for facial application, as shown in Table 14. All formulations exhibited a uniform cream texture, homogeneous color, and good physical stability, with a consistent pH of 5.5 ± 0.0, which is appropriate for skin application [81]. The inclusion of foundation pigments resulted in an increase in formulation viscosity. With respect to sun protection efficacy, formulations without sunscreen agents exhibited an SPF of 0 and a PPD/UVA-PF of 12, corresponding to a PA+++ rating. In contrast, formulations containing sunscreen agents demonstrated markedly higher photoprotective performance, which was further enhanced by the presence of foundation pigments. The TMO-based sunscreen cream containing foundation pigments exhibited the highest SPF (43.09) and a PPD/UVA-PF of 24.37, achieving a PA++++ rating. By comparison, the olive oil-based formulation showed an SPF of 27.53 and a PPD/UVA-PF of 17.81, also with a PA++++ rating. In addition, blue light protection was higher in the TMO-based formulations than in those containing olive oil. These results indicate that TMO possesses strong potential for use in sunscreen products, offering superior photoprotective performance compared with commonly used vegetable oils such as olive oil. Furthermore, the TMO-based sunscreen cream without foundation pigments is suitable for both facial and body application, whereas the pigmented TMO-based sunscreen cream is particularly well suited for facial use.

## 4. Conclusions

This research presents the potential of cricket-derived oils, with particular emphasis on TMO, as innovative functional ingredients for sunscreen and skin-protective cosmetic formulations. The findings demonstrated that cricket oil can be effectively integrated into sunscreen systems containing both physical and chemical UV filters without compromising formulation stability. Rather than acting solely as a lipid base, TMO provides added functional value by supporting biological protection mechanisms that complement conventional photoprotective strategies. The incorporation of TMO into sunscreen formulations contributed to a more comprehensive approach to skin protection, addressing not only UV radiation but also oxidative stress and high-energy visible light exposure. Its compatibility with established sunscreen agents enables the development of formulations with enhanced broad-spectrum performance while potentially reducing the reliance on high concentrations of chemical UV filters. This aligns with current trends in cosmetic science toward safer, milder, and more sustainable product designs. The findings support the use of TMO as a novel and sustainable alternative to traditional plant oils in sunscreen and cosmeceutical applications. Its multifunctional role, favorable safety profile, and ability to enhance sunscreen efficacy highlighted its promise for further product development and commercialization. Future studies may focus on in vivo performance and consumer acceptance to fully establish the practical potential of cricket oil-based sunscreen products.

## Figures and Tables

**Table 1:** Cold-process (CP) cream base formulations.

Ingredient	Amount (% *w*/*w*)									
	CP1	CP2	CP3	CP4	CP5	CP6	CP7	CP8	CP9	CP10
DI water q.s.	100	100	100	100	100	100	100	100	100	100
Disodium EDTA	0.1	0.1	0.1	0.1	0.1	0.1	0.1	0.1	0.1	0.1
Glycerin	4	4	4	4	4	4	4	4	4	4
Butylene glycol	3	3	3	3	3	3	3	3	3	3
Spectrastat™ BHL	1	1	1	1	1	1	1	1	1	1
Cricket oil	1	2	3	4	5	1	2	3	4	5
Myritol^®^ 318	3	3	3	3	3	3	3	3	3	3
LipidSoft^TM^ Lite	1	1	1	1	1	1	1	1	1	1
Cream Maker^TM^ XL	7	7	7	7	7	7	7	7	7	7
Novemer^TM^ EC-2	2	2	2	2	2	4	4	4	4	4

Note: INCI names of each ingredient are as follows: Spectrastat™ BHL: caprylhydroxamic acid (and) 1,2-hexanediol (and) butylene glycol; Cricket oil: *Teleogryllus mitratus* oil; Myritol^®^ 318: caprylic/capric triglyceride; LipidSoft™ Lite: isononyl isononanoate; Cream Maker™ XL: cyclomethicone, PEG-10 dimethicone, lauryl PEG-9 polydimethylsiloxyethyl dimethicone, and disteardimonium hectorite; Novemer™ EC-2: sodium acrylates/beheneth-25 methacrylate crosspolymer (and) hydrogenated polydecene (and) lauryl glucoside.

**Table 2:** Hot-process (HP) cream base formulations.

Ingredient	Amount (% *w*/*w*)				
	HP1	HP2	HP3	HP4	HP5
DI water q.s.	100	100	100	100	100
Disodium EDTA	0.1	0.1	0.1	0.1	0.1
Glycerin	4	4	4	4	4
Butylene glycol	3	3	3	3	3
Xanthan gum	0.1	0.1	0.1	0.1	0.1
Carbomer^®^ U21	0.1	0.1	0.1	0.1	0.1
Triethanolamine	0.2	0.2	0.2	0.2	0.2
Cricket oil	1	2	3	4	5
Myritol^®^ 318	3	3	3	3	3
LipidSoft^TM^ Lite	1	1	1	1	1
Lexemul 561	5	5	5	5	5
Oil blender^TM^	1	1	1	1	1
Wax C	0.5	1	1.5	2	2.5
Vitamin E	0.1	0.1	0.1	0.1	0.1
Spectrastat^TM^ BHL	1	1	1	1	1
Beige EasyMix^TM^	0.5	0.5	0.5	0.5	0.5
Titanium Dioxide	0.5	0.5	0.5	0.5	0.5
Dimethicone	1	1	1	1	1

Note: INCI names of each ingredient are as follows: Carbomer^®^ U21: acrylates/C10-30 alkyl acrylate crosspolymer; Cricket oil: *Teleogryllus mitratus* oil; Myritol^®^ 318: caprylic/capric triglyceride; LipidSoft™ Lite: isononyl isononanoate; Lexemul 561: glyceryl stearate and PEG-100 stearate; Oil Blender™: polyglyceryl-3 diisostearate; Wax C: cetyl alcohol; Vitamin E: tocopherol; Spectrastat™ BHL: caprylhydroxamic acid (and) 1,2-hexanediol (and) butylene glycol; Beige EasyMix™: Mica, iron oxides (red, CI 77491), iron oxides (yellow, CI 77492), dimethicone.

**Table 3:** Composition of cricket oil-based sunscreen formulations prepared by hot process (HP) containing physical (PHP) or chemical (CHP) UV filters.

Ingredient	Amount (% *w*/*w*)									
	PHP1	PHP2	PHP3	PHP4	CHP1	CHP2	CHP3	CHP4	CHP5	CHP6
DI water q.s.	100	100	100	100	100	100	100	100	100	100
Disodium EDTA	0.1	0.1	0.1	0.1	0.1	0.1	0.1	0.1	0.1	0.1
Glycerin	4	4	4	4	4	4	4	4	4	4
Butylene glycol	3	3	3	3	3	3	3	3	3	3
Xanthan Gum	0.1	0.1	0.1	0.1	0.1	0.1	0.1	0.1	0.1	0.1
Carbomer U21	0.1	0.1	0.1	0.1	0.1	0.1	0.1	0.1	0.1	0.1
Triethanolamine	0.2	0.2	0.2	0.2	0.2	0.2	0.2	0.2	0.2	0.2
Cricket oil	3	3	3	3	3	3	3	3	3	3
Myritol^®^ 318	3	3	3	3	3	3	3	3	3	3
LipidSoft^TM^ Lite	1	1	1	1	1	1	1	1	1	1
Lexemul 561	5	5	5	5	5	5	5	5	5	5
Oil blender^TM^	1	1	1	1	1	1	1	1	1	1
Wax C	1.5	1.5	1.5	1.5	1.5	1.5	1.5	1.5	1.5	1.5
Dimethicone	1	1	1	1	1	1	1	1	1	1
Vitamin E	0.1	0.1	0.1	0.1	0.1	0.1	0.1	0.1	0.1	0.1
Spectrastat^TM^ BHL	1	1	1	1	1	1	1	1	1	1
Zinc oxide	-	3	6	9	-	-	-	-	-	3
Titanium dioxide	9	6	3	-	-	-	-	-	-	6
Octisalate	-	-	-	-	-	1	3	5	5	-
Avobenzone	-	-	-	-	3	3	1	-	3	3

Note: INCI names of each ingredient are as follows: Carbomer^®^ U21: acrylates/C10-30 alkyl acrylate crosspolymer; Cricket oil: *Teleogryllus mitratus* oil; Myritol^®^ 318: caprylic/capric triglyceride; LipidSoft™ Lite: isononyl isononanoate; Lexemul 561: glyceryl stearate and PEG-100 stearate; Oil Blender™: polyglyceryl-3 diisostearate; Wax C: cetyl alcohol; Vitamin E: tocopherol; Spectrastat™ BHL: caprylhydroxamic acid (and) 1,2-hexanediol (and) butylene glycol; Beige EasyMix™: Mica, iron oxides (red, CI 77491), iron oxides (yellow, CI 77492), dimethicone.

**Table 4:** Composition of cricket oil-based sunscreen formulations containing physical and chemical UV filters.

Ingredient	Amount (% *w*/*w*)								
	Blank	COB	COS	OOB	OOS	COBP	COSP	OOBP	OOSP
DI water q.s.	100	100	100	100	100	100	100	100	100
Disodium EDTA	0.1	0.1	0.1	0.1	0.1	0.1	0.1	0.1	0.1
Glycerin	4	4	4	4	4	4	4	4	4
Butylene glycol	3	3	3	3	3	3	3	3	3
Xanthan Gum	0.1	0.1	0.1	0.1	0.1	0.1	0.1	0.1	0.1
Carbomer U21	0.1	0.1	0.1	0.1	0.1	0.1	0.1	0.1	0.1
Triethanolamine	0.2	0.2	0.2	0.2	0.2	0.2	0.2	0.2	0.2
Cricket oil	-	3	3	-	-	3	3	-	-
Olive oil	-	-	-	3	3	-	-	3	3
Myritol^®^ 318	3	3	3	3	3	3	3	3	3
LipidSoft^TM^ Lite	1	1	1	1	1	1	1	1	1
Lexemul 561	5	5	5	5	5	5	5	5	5
Oil blender^TM^	1	1	1	1	1	1	1	1	1
Wax C	1.5	1.5	1.5	1.5	1.5	1.5	1.5	1.5	1.5
Dimethicone	1	1	1	1	1	1	1	1	1
Vitamin E	0.1	0.1	0.1	0.1	0.1	0.1	0.1	0.1	0.1
Spectrastat^TM^ BHL	1	1	1	1	1	1	1	1	1
Zinc oxide	-	-	3	-	3	-	3	-	3
Titanium dioxide	-	-	6	-	6	-	6	-	6
Octisalate	-	-	5	-	5	-	5	-	5
Avobenzone	-	-	3	-	3	-	3	-	3
Beige EasyMix^TM^	-	-	-	-	-	0.1	0.1	0.1	0.1

Note: INCI names of each ingredient are as follows: Carbomer^®^ U21: acrylates/C10-30 alkyl acrylate crosspolymer; Cricket oil: *Teleogryllus mitratus* oil; Myritol^®^ 318: caprylic/capric triglyceride; LipidSoft™ Lite: isononyl isononanoate; Lexemul 561: glyceryl stearate and PEG-100 stearate; Oil Blender™: polyglyceryl-3 diisostearate; Wax C: cetyl alcohol; Vitamin E: tocopherol; Spectrastat™ BHL: caprylhydroxamic acid (and) 1,2-hexanediol (and) butylene glycol; Beige EasyMix™: Mica, iron oxides (red, CI 77491), iron oxides (yellow, CI 77492), dimethicone; Beige EasyMix™: Mica, iron oxides (red, CI 77491), iron oxides (yellow, CI 77492), dimethicone. Blank refers to the formulation without any oil or UV filters; COB refers to the TMO-based cream formulation; COS refers to the TMO-based sunscreen cream formulation; OOB refers to the olive oil-based cream formulation; OOS refers to the olive oil-based sunscreen cream formulation; COBP refers to the TMO-based cream formulation containing foundation pigments; COSP refers to the TMO-based sunscreen cream formulation containing foundation pigments; OOBP refers to the olive oil-based cream formulation containing foundation pigments; OOSP refers to the olive oil-based sunscreen cream formulation containing foundation pigments.

**Table 5:** Yield of dried crickets and cricket oil.

Crickets	Hot-Air Drying Process			Freeze-Drying Process [27]		
	Yield (% *w*/*w* Based on Fresh Weight)	Cricket Oil (% by Weight)		Yield (% *w*/*w* Based on Fresh Weight)	Cricket Oil (% by Weight)	
		Based on Dry Weight	Based on Fresh Weight		Based on Dry Weight	Based on Fresh Weight
*G. bimaculatus*	30.2	17.9	5.4	27.6	24.6	6.8
*T. mitratus*	28.9	14.3	4.1	28.9	21.0	6.1
*A. domesticus*	28.7	13.7	3.9	27.6	15.5	4.3

**Table 6:** Fatty acid composition of cricket oil in comparison with olive oil.

Fatty Acids		Fatty Acid Content (%)			
		Olive Oil	GBO	TMO	ADO
Saturated fat					
Palmitic acid	(C16:0)	12.94 ± 0.07	26.11 ± 0.05	25.81 ± 0.01	26.25 ± 0.06
Stearic acid	(C18:0)	3.37 ± 0.00	7.80 ± 0.01	9.18 ± 0.01	6.73 ± 0.03
Myristic acid	(C14:0)	-	2.90 ± 0.01	-	0.72 ± 0.01
Lauric acid	(C12:0)	-	1.07 ± 0.01	-	0.04 ± 0.00
Arachidic acid	(C20:0)	0.44 ± 0.03	0.53 ± 0.00	0.67 ± 0.01	0.33 ± 0.00
Margaric acid	(C17:0)	-	0.16 ± 0.01	-	0.31 ± 0.01
Pentadecanoic acid	(C15:0)	-	-	-	0.15 ± 0.01
Behenic acid	(C22:0)	0.12 ± 0.00	-	0.43 ± 0.01	-
Unsaturated fatty acid					
Monounsaturated fatty acid					
Oleic acid	(C18:1)	73.92 ± 0.05	29.90 ± 0.00	30.56 ± 0.01	31.70 ± 0.11
Palmitoleic acid	(C16:1)	1.12 ± 0.01	1.30 ± 0.01	0.36 ± 0.01	0.45 ± 0.02
Cis-11-eicosenoic acid	(C20:1)	0.24 ± 0.01	-	0.04 ± 0.01	0.11 ± 0.00
Myristoleic acid	(C14:1)	-	0.14 ± 0.01	-	-
Erucic acid	(C22:1)	-	-	-	0.22 ± 0.03
Polyunsaturated fatty acid					
Linoleic acid	(C18:2)	7.21 ± 0.02	29.35 ± 0.05	29.33 ± 0.01	31.27 ± 0.01
Linolenic acid	(C18:3)	0.66 ± 0.02	0.77 ± 0.01	1.15 ± 0.01	0.82 ± 0.00
Arachidonic acid	(C20:4)	-	-	-	0.25 ± 0.01
Cis-8,11,14-eicosatrienoic acid	(C20:3)	-	-	0.14 ± 0.01	0.20 ± 0.01
Cis-11,14,17-eicosatrienoic acid	(C20:3)	-	-	0.12 ± 0.01	0.14 ± 0.00

Note: Different cricket oils were evaluated, including *Gryllus bimaculatus* (GBO), *Teleogryllus mitratus* (TMO), and *Acheta domesticus* (ADO).

**Table 7:** Chorioallantoic membrane (CAM) response to exposure to the test substance, irritation score, and irritation classification.

Duration (min)	Positive Control	Negative Control	GBO	TMO	ADO
**0**	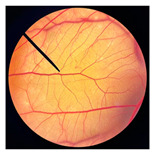	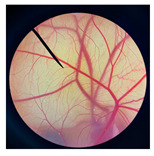	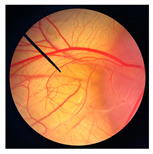	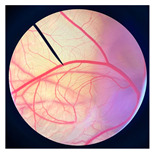	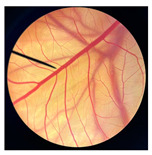
**5**	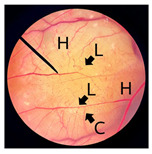	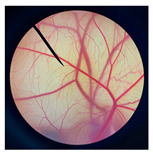	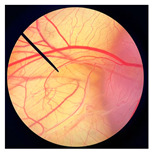	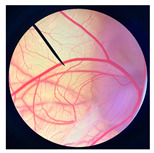	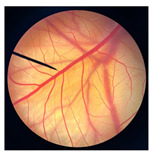
**60**	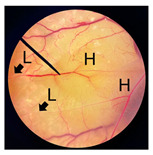	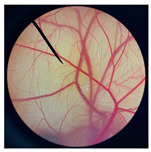	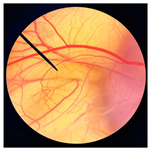	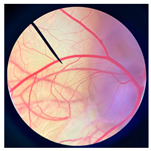	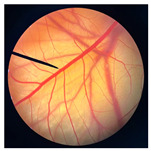
Irritation score	18.5 ± 0.1 ^a^	0.0 ± 0.0 ^b^	0.0 ± 0.0 ^b^	0.0 ± 0.0 ^b^	0.0 ± 0.0 ^b^
Irritation potential	Severe irritation	No irritation	No irritation	No irritation	No irritation

Note: The positive control was a 1% *w*/*v* sodium lauryl sulfate solution, and the negative control was a 0.9% *w*/*v* sodium chloride solution. Different cricket oils were evaluated, including *Gryllus bimaculatus* (GBO), *Teleogryllus mitratus* (TMO), and *Acheta domesticus* (ADO). Different letters (a and b) denote statistically significant differences among groups as determined by one-way ANOVA followed by Tukey’s post hoc test (*p* < 0.05). The letters H, L, and C denote hemorrhage, lysis, and coagulation, respectively.

**Table 8:** SPF, PPD/UVA-PF, and blue light protection of cricket oils.

Oils	SPF	PPD/UVA-PF	Blue Light Protection (%)
Olive oil	0.00 ± 0.00 ^b^	-	0.0 ± 0.0 ^b^
GBO	0.00 ± 0.00 ^b^	-	0.0 ± 0.0 ^b^
TMO	0.04 ± 0.00 ^a^	12.1 (PA+++)	1.2 ± 0.0 ^a^
ADO	0.00 ± 0.00 ^b^	-	0.0 ± 0.0 ^b^

Note: Sun Protection Factor (SPF) indicates the ability to protect against UVB radiation. Persistent pigment darkening/Ultraviolet A protection factor (PPD/UVA-PF) indicates protection against UVA radiation. Blue light protection indicates the ability to protect against blue light. Different cricket oils were evaluated, including *Gryllus bimaculatus* (GBO), *Teleogryllus mitratus* (TMO), and *Acheta domesticus* (ADO). Different letters (a and b) denote statistically significant differences among groups as determined by one-way ANOVA followed by Tukey’s post hoc test (*p* < 0.05).

**Table 9:** Physical appearance and stability of cold-processed cream formulations.

Novemer^TM^ EC-2 (% *w*/*w*)	TMO Content (% *w*/*w*)				
	1	2	3	4	5
**2**	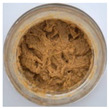	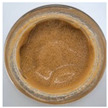	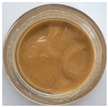	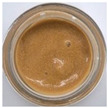	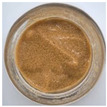
Stability	Unstable	Unstable	Unstable	Unstable	Unstable
**4**	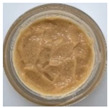	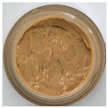	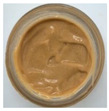	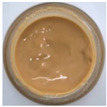	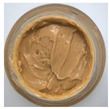
Stability	Stable	Stable	Stable	Stable	Stable
pH	5.5 ± 0.0	5.5 ± 0.0	5.5 ± 0.0	5.5 ± 0.0	5.5 ± 0.0
Viscosity (Pa.s)	1.04 ± 0.1 ^a^	2.01 ± 0.1 ^b^	2.99 ± 0.1 ^c^	3.47 ± 0.1 ^d^	3.83 ±0.1 ^e^

Note: The formulation was developed with oil from *Teleogryllus mitratus* (TMO). Different letters (a–e) denote statistically significant differences among groups as determined by one-way ANOVA followed by Tukey’s post hoc test (*p* < 0.05).

**Table 10:** Physical appearance and stability of hot-processed cream formulations.

Characteristics	TMO Content (% *w*/*w*)				
	1	2	3	4	5
Physical appearance	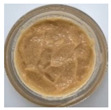	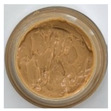	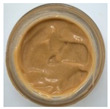	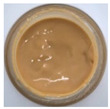	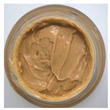
Stability	Stable	Stable	Stable	Stable	Stable
pH	5.5 ± 0.0	5.5 ± 0.0	5.5 ± 0.0	5.5 ± 0.0	5.5 ± 0.0
Viscosity (Pa.s)	1.74 ± 0.01 ^a^	2.94 ± 0.01 ^b^	2.99 ± 0.01 ^b^	3.47 ± 0.01 ^c^	3.40 ± 0.01 ^c^

Note: The formulation was developed with oil from *Teleogryllus mitratus* (TMO). Different letters (a–c) denote statistically significant differences among groups as determined by one-way ANOVA followed by Tukey’s post hoc test (*p* < 0.05).

**Table 11:** Physical appearance, stability, and sun protection performance of cricket oil-based creams formulated with physical sunscreen agents.

Characteristics	Zinc Oxide:Titanium Dioxide (%*w*/*w*)			
	0:9	3:6	6:3	9:0
Physical appearance	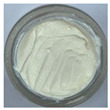	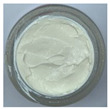	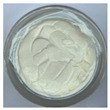	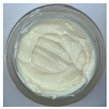
Stability	Stable	Stable	Stable	Stable
pH	5.5 ± 0.0	5.5 ± 0.0	5.5 ± 0.0	5.5 ± 0.0
Viscosity (Pa.s)	1.40 ± 0.01 ^c^	0.79 ± 0.01 ^a^	0.80 ± 0.01 ^a^	0.90 ± 0.01 ^b^
SPF	0.22	33.41	29.41	16.27
PPD/UVA-PF	12.00	24.00	23.50	18.90
PA	+++	++++	++++	++++
Blue light protection (%)	0.0	33.8	33.0	25.1

Note: The formulation was developed with oil from *Teleogryllus mitratus* (TMO). PA+++ indicates a PPD value of 8 to <16 (high UVA protection), and PA++++ indicates a PPD value ≥16 (very high UVA protection). Different letters (a–c) denote statistically significant differences among groups as determined by one-way ANOVA followed by Tukey’s post hoc test (*p* < 0.05).

**Table 12:** Physical appearance, stability, and sun protection performance of cricket oil-based creams formulated with chemical sunscreen agents.

Characteristics	Octisalate:Avobenzone				
	0:3	1:3	3:1	5:0	5:3
Physical appearance	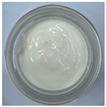	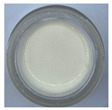	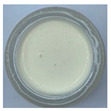	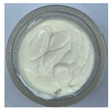	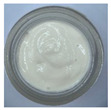
Stability	Stable	Stable	Stable	Stable	Stable
pH	5.5 ± 0.0	5.5 ± 0.0	5.5 ± 0.0	5.5 ± 0.0	5.5 ± 0.0
Viscosity (Pa.s)	0.97 ± 0.02 ^e^	0.56 ± 0.02 ^c^	0.32 ± 0.02 ^a^	0.60 ± 0.01 ^d^	0.51 ± 0.01 ^b^
SPF	14.31	17.02	23.28	19.86	40.51
PPD/UVA-PF	22.53	22.01	22.85	22.04	39.17
PA	++++	++++	++++	++++	++++
Blue light protection (%)	26.1	30.2	31.6	29.8	34.0

Note: The formulation was developed with oil from *Teleogryllus mitratus* (TMO). PA++++ indicates a PPD value ≥16 (very high UVA protection). Different letters (a–e) denote statistically significant differences among groups as determined by one-way ANOVA followed by Tukey’s post hoc test (*p* < 0.05).

**Table 13:** Physical appearance, stability, and sun protection performance of cricket oil-based creams formulated with physical and chemical sunscreen agents.

Characteristics	Sunscreen Formulations				
	Blank	COB	COS	OOB	OOS
Physical appearance	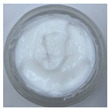	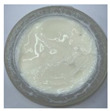	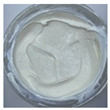	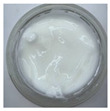	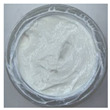
Stability	Stable	Stable	Stable	Stable	Stable
pH	5.5 ± 0.0	5.5 ± 0.0	5.5 ± 0.0	5.5 ± 0.0	5.5 ± 0.0
Viscosity (Pa.s)	0.91 ± 0.01 ^a^	0.99 ± 0.01 ^b^	1.29 ± 0.01 ^c^	0.98 ± 0.01 ^b^	1.25 ± 0.01 ^c^
SPF	0.00	0.00	40.20	0.00	36.36
PPD/UVA-PF	12.00	12.00	22.83	12.00	21.70
PA	+++	+++	++++	+++	++++
Blue light protection (%)	0.2	0.2	32.1	0.2	30.0

Note: Blank refers to the formulation without olive oil or *Teleogryllus mitratus* oil (TMO). COB refers to the TMO-based cream formulation; COS refers to the TMO-based sunscreen cream formulation; OOB refers to the olive oil-based cream formulation; OOS refers to the olive oil-based sunscreen cream formulation. PA+++ indicates a PPD value of 8 to <16 (high UVA protection), and PA++++ indicates a PPD value ≥16 (very high UVA protection). Different letters (a–c) denote statistically significant differences among groups as determined by one-way ANOVA followed by Tukey’s post hoc test (*p* < 0.05).

**Table 14:** Physical appearance, stability, and sun protection performance of cricket oil-based creams formulated with physical sunscreens, chemical sunscreens, and foundation pigments.

Characteristics	Sunscreen Formulations			
	COBP	COSP	OOBP	OOSP
Physical appearance	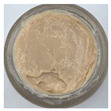	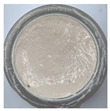	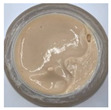	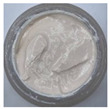
Stability	Stable	Stable	Stable	Stable
pH	5.5 ± 0.0	5.5 ± 0.0	5.5 ± 0.0	5.5 ± 0.0
Viscosity (Pa.s)	0.91 ± 0.01 ^a^	1.34 ± 0.01 ^b^	0.95 ± 0.01 ^a^	1.37 ± 0.01 ^b^
SPF	0.00	43.09	0.00	27.53
PPD/UVA-PF	12.00	24.37	12.00	17.81
PA	+++	++++	+++	++++
Blue light protection (%)	0.6	35.1	0.5	21.0

Note: The formulation was developed with oil from *Teleogryllus mitratus* (TMO). COBP refers to the TMO-based cream formulation containing foundation pigments; COSP refers to the TMO-based sunscreen cream formulation containing foundation pigments; OOBP refers to the olive oil-based cream formulation containing foundation pigments; OOSP refers to the olive oil-based sunscreen cream formulation containing foundation pigments. PA+++ indicates a PPD value of 8 to <16 (high UVA protection), and PA++++ indicates a PPD value ≥16 (very high UVA protection). Different letters (a and b) denote statistically significant differences among groups as determined by one-way ANOVA followed by Tukey’s post hoc test (*p* < 0.05).

## Data Availability

The datasets generated and analyzed during the current study are available from the corresponding author upon request.

## Abbreviations

The following abbreviations are used in this manuscript:

AAD	American Academy of Dermatology
ADO	*Acheta domesticus* oil
ANOVA	Analysis of variance
BF_3_	Boron trifluoride
CAM	Chorioallantoic membrane
CDA	Canadian Dermatology Association
CHP	Chemical sunscreen formulation
CO_2_	Carbon dioxide
COB	Cricket oil-based cream formulation
COBP	Cricket oil-based cream formulation containing foundation pigments
COS	Cricket oil-based sunscreen cream formulation
COSP	Cricket oil-based sunscreen cream formulation containing foundation pigments
CP	Cold-process formulation
DCFH-DA	2′,7′-Dichlorodihydrofluorescein diacetate
DI	Deionized
DMEM	Dulbecco’s Modified Eagle Medium
DMSO	Dimethyl sulfoxide
EDTA	Ethylenediaminetetraacetic acid
FAME	Fatty acid methyl ester
FBS	Fetal bovine serum
GC–MS	Gas chromatography–mass spectrometry
GBO	*Gryllus bimaculatus* oil
GMP	Good Manufacturing Practice
H_2_O_2_	Hydrogen peroxide
HACCP	Hazard Analysis and Critical Control Points
HET-CAM	Hen’s egg test on the chorioallantoic membrane
HP	Hot-process formulation
INCI	International nomenclature of cosmetic ingredients
IS	Irritation score
L-NAME	N-nitro-L-arginine methyl ester
LPS	Lipopolysaccharide
MTT	3-(4,5-Dimethylthiazol-2-yl)-2,5-diphenyltetrazolium bromide
MUFAs	Monounsaturated fatty acids
NHDF	Normal human dermal fibroblasts
NO^•^	Nitric oxide
NOS	Nitric oxide synthase
NSS	Normal saline solution
O/W	Oil-in-water
OO	Olive oil
OOB	Olive oil-based cream formulation
OOBP	Olive oil-based cream formulation containing foundation pigments
OOS	Olive oil-based sunscreen cream formulation
OOSP	Olive oil-based sunscreen cream formulation containing foundation pigments
PA	Protection grade of UVA
PBS	Phosphate-buffered saline
PHP	Physical sunscreen formulation
PPD	Persistent pigment darkening
PUFAs	Polyunsaturated fatty acids
RAW 264.7	Murine macrophage cell line
RNS	Reactive nitrogen species
ROS	Reactive oxygen species
SFAs	Saturated fatty acids
SLS	Sodium lauryl sulfate
SPF	Sun protection factor
TEWL	Transepidermal water loss
TMO	*Teleogryllus mitratus oil*
Trolox	6-Hydroxy-2,5,7,8-tetramethylchroman-2-carboxylic acid
UVA	Ultraviolet A radiation
UVB	Ultraviolet B radiation
UVA-PF	Ultraviolet A protection factor
